# The PI3K/Akt pathway upregulates Id1 and integrin α4 to enhance recruitment of human ovarian cancer endothelial progenitor cells

**DOI:** 10.1186/1471-2407-10-459

**Published:** 2010-08-26

**Authors:** Yajuan Su, Lei Zheng, Qian Wang, Jie Bao, Zhen Cai, Ailan Liu

**Affiliations:** 1 Department of Clinical Laboratory, Nanfang Hospital, Southern Medical University, Guangzhou, China

## Abstract

**Background:**

Endothelial progenitor cells (EPCs) contribute to tumor angiogenesis and growth. We aimed to determine whether inhibitors of differentiation 1 (Id1) were expressed in circulating EPCs of patients with ovarian cancer, whether Id1 could mediate EPCs mobilization and recruitment, and, if so, what underlying signaling pathway it used.

**Methods:**

Circulating EPCs cultures were from 25 patients with ovarian cancer and 20 healthy control subjects. Id1 and integrin α4 expression were analyzed by real-time reverse transcription-polymerase chain reaction and western blot. EPCs proliferation, migration, and adhesion were detected by MTT, transwell chamber, and EPCs-matrigel adhesion assays. Double-stranded DNA containing the interference sequences were synthesized according to the structure of a pGCSIL-GFP viral vector and then inserted into a linearized vector. Positive clones were identified as lentiviral vectors that expressed human Id1 short hairpin RNA (shRNA).

**Results:**

Id1 and integrin α4 expression were increased in EPCs freshly isolated from ovarian cancer patients compared to those obtained from healthy subjects. siRNA-mediated Id1 downregulation substantially reduced EPCs function and integrin α4 expression. Importantly, Inhibition of PI3K/Akt inhibited Id1 and integrin α4 expression, resulting in the decreasing biological function of EPCs.

**Conclusions:**

Id1 induced EPCs mobilization and recruitment is mediated chiefly by the PI3K/Akt signaling pathway and is associated with activation of integrin α4.

## Background

Numerous studies have indicated that angiogenesis, a process mediated by endothelial progenitor cells (EPCs) derived from the bone marrow, is increased in many tumors due to elevated levels of angiogenic factors in the peripheral blood. An increase in EPCs supply and mobilization from the bone marrow can accelerate tumor angiogenesis [1-3]. A number of reports have described the incorporation of EPCs into tumor vessels in both tumor models and human patients. However the mechanisms that govern the behavior of EPCs, from their origin in the BM to their release into the circulation in response to pro-angiogenic stimuli, are still poorly understood [4,5].

Id1 is a member of a family of 4 proteins (Id1-4) known to inhibit the activity of basic helix loop helix transcription factors by blocking their ability to bind DNA [6]. Loss of Id1 in the BM leads to a complete loss of EPCs in peripheral blood, which has been correlated with a block in tumor neovascularization and delayed tumor growth [7]. However, the actual role of Id1 in regulating EPCs mobilization or recruitment remains unknown. Given the key roles that EPCs migration and adhesion may play in tumor metastasis, we tried to investigate the effect of Id1 on circulating EPCs mobilization and recruitment and the possible signal transduction pathways involved in the process.

We knocked down the expression of Id1 by an siRNA-mediated Id1 lentiviral construct to determine the functional importance of Id1 in EPCs of patients with ovarian cancer,. Our results indicate that Id1 contributes to the migration and adhesion of EPCs in ovarian cancer patients and that Id1 may be important in the pathogenesis of ovarian cancer. Next, we evaluated the effects of inhibiting the phosphatidylinositol 3-kinase (PI3K)/Akt signaling pathway on Id1 and integrin α4 in EPCs of patients with ovarian cancer.

The identification of Id1 as a common target gene in EPCs migration and adhesion suggested that Id1 might serve as a novel therapeutic target in ovarian cancer. Id1 is expressed in bone marrow-derived EPCs [8] and is highly expressed in ovarian cancer cells [9,10]. Inhibiting Id1 can therefore both disrupt ovarian cancer cells growth and prevent blood vessels from feeding the ovarian cancer cells.

## Methods

### Patients

This study was approved by the local ethics committee in China and informed consent was obtained from all study participants. Twenty-five patients (median age, 41 years old; age range, 21-59 years old) with histologically proven ovarian cancer, including serous cancer (n = 14), mucinous cancer (n = 7), and endometrioid cancer (n = 4), were studied along with a control group of healthy women (n = 20, age range, 18-35 years old). These diagnosed ovarian cancer patients had no additional malignant, inflammatory, or ischemic disease; wounds; or ulcers that could influence the number of EPCs.

### EPCs isolation and characterization

Total MNCs were isolated from 20 ml human peripheral blood samples from ovarian cancer patients and healthy women by density gradient centrifugation with Histopaque-1077 (density 1.077 g/ml; Sigma). MNCs were plated in 1 ml endothelial growth medium (EGM-2; Lonza) on fibronectin-coated (Sigma) twenty-four-well plates. After 24 h of culturing, unattached cells were discarded and attached cells were cultured as before. Medium was replaced every 2 days thereafter, and each colony/cluster was followed up. After 7 days in culture, colony forming cells were recognized as attached spindle-shaped cells. The adherent cells were incubated with DiI-acLDL (Molecule Probes) and then fixed in 2% paraformaldehyde and counterstained with fluorescein isothiocyanate (FITC)-labeled lectin from ulex europaeus agglutinin (UEA-1) (Sigma). The fluorescent images were recorded under a fluorescent microscope. Cells were also characterized by immunofluorescence staining for von Willebrand factor (vWF) and expression of CD31 and VEGFR2 (Becton Dickinson).

### Quantitative real-time RT-PCR

Total RNA isolation and cDNA synthesis from cultured EPCs were performed using Trizol and the SuperScript II Reverse Transcriptase kit (Invitrogen, USA) according to the manufacturer's instructions.

Real-time PCR was performed with the Mx3000p Real Time PCR System (Stratagene, USA) using the following thermal cycling conditions: 10 sec at 95°C followed by 40 cycles of 15 sec at 95°C, 20 sec at 60°C, and 7 sec at 72°C. SYBR^® ^GreenER qPCR SuperMix Universal S (Invitrogen, USA) (25 μl) were performed in triplicate. A no-template control (replacing RNA with water) was used as a negative control. Id1 and integrin α4 mRNA in the EPCs was determined by relative quantitation, interpolating from a standard curve of template DNA of known concentration and then normalized using β-actin as an internal control. Data were analyzed by 2^-ΔΔCt^.

The primer sequences used for real-time PCR were as follows: Id1, 5-GTAAACGTGCTGCTCTACGACATGA-3 and 5-AGCTCCAACTGAAGGTCCCTGA-3; integrin α4, 5-TTGACAACAACGGTACTGCTAC-3 and 5-TGGTGAACACTGTGCTGATTAC-3; and β-actin, 5-TGGCACCCAGCACAATGAA-3 and 5-CTAAGTCATAGTCCGCCTAGAAGCA-3.

### Western blots

The EPCs were collected with sample buffer. Cell lysates were centrifuged at 10000 *g *for 10 min at 4°C and the supernatant was stored at -70°C. Protein concentrations were determined with a Bio-Rad kit. 50-μg aliquots of protein were subjected to 12% and 6% SDS-PAGE gels. Then the protein was blotted onto a PVDF membrane. Primary antibodies against Id1(1:1000, Becton Dickinson), integrin α4 (1:1000, Becton Dickinson), Phospho-Akt (ser473) (1:1000, Cell Signaling), Total-Akt (1:2000, Cell Signaling), and β-actin (1:2000, Becton Dickinson) were used according to the manufacturer's recommendations. After washing the membrane, a second antibody (HRP-conjugated anti-mouse IgG) (1:2000, Becton Dickinson) was used to detect Id1, integrin α4, Phospho-Akt, Total-Akt, and β-actin. The bands were visualized using the ECL detection system (Pierce) with 5 to 30 min exposure after washing the membrane. β-actin were used as protein loading controls.

### Construction of lentiviral vector expressing Id1-specific shRNA

Three different Id1-specific target sequences were chosen according to online siRNA tools provided by Invitrogen (http://www.invitrogen.com/rnai) using the Id1 reference sequence (Gene Bank Accession No NM_002165). Double-stranded DNA containing the interference sequences were synthesized according to the structure of a pGCSIL-GFP viral vector (Gikai gene company, Shanghai, China) and then inserted into a linearized vector. All the constructs were cloned and sequenced to confirm their structure. The positive clones were identified as lentiviral vectors that expressed human Id1 short hairpin RNA (shRNA), hereafter designated pGCSIL/Id1-1, pGCSIL/Id1-2, and pGCSIL/Id1-3. The three lentiviral vectors were transfected into HEK 293 cells separately to evaluate their RNA interference effects and pGCSIL/Id1-3 (sequence: 5'-gaCATGAACGGCTGTTACTCA-3') induced the highest levels of downregulation. So pGCSIL/Id1-3 vector and viral packaging system (Gikai gene company, Shanghai, China, containing an optimized mixture of two packaging plasmids: pHelper 1.0 vector and pHelper 2.0 vector) were cotransfected into 293 cells to replicate competent lentivirus. Viral supernatant was harvested 48 h after transfection, filtered through a 0.45-mm cellulose acetate filter and frozen at -70°C. The lentivirus (LV) containing the human Id1 shRNA-expressing cassette (pGCSIL/Id1-3) was used as a positive control for lentivirus production and denoted as Id1-RNAi-LV in the next experiments. The pGCSIL/U6 mock vector was also packaged and used as a negative control, denoted as NC-GFP-LV. Viral concentrations were determined by serial dilutions of the concentrated vector stocks in 293 cells in 96-well plates. The number of green fluorescent protein (GFP)-positive cells was measured 4 d post-transduction under microscopy. The titers averaged 2 × 10^9 ^TU/mL.

### In vitro transduction of EPCs

For lentiviral transduction, the primary EPCs were passaged into 6-well plates at a density of 1 × 10^5 ^cells/well. When cells reached 30% confluence (typically on the third day after subculturing) the medium was replaced with 1 mL of fresh medium containing lentivirus at an MOI of 150 and 6 μg/mL polybrene (Gikai gene company, Shanghai, China). The medium was replaced with fresh medium on the following day. Five days after transduction cells were analyzed by flow cytometry using a BD FACSCalibur cell analyzer (BD Biosciences). The percentage of GFP-positive cells and mean fluorescence intensity (MFI) of GFP-positive cells were determined with WinMDI 2.8 software (J. Trotter, Flow Cytometry Core Facility, Scripps Research Institute, La Jolla, CA). Means and standard deviations from experiments performed in triplicate are given.

### Proliferation assay

The 3-(4,5-dimethylthiazol-2-yl)-2,5-diphenyltetrazolium bromide (MTT) assay was used to determine EPCs proliferation. EPCs were incubated with 50-400 μg/ml AGE-albumin or 400 μg/ml unmodified albumin for 24 h, then supplemented with MTT (0.5 mg/ml, Sigma) and incubated for a further 4 h. The blue formazan thus produced was solubilized with dimethyl sulfoxide and absorbance was measured at 550-650 nm.

### Transwell Migration Assay

After 7 days of incubation, culture medium was removed and replaced with EBM-2 without any supplement 12 hours before the migration assay. EPCs migration was evaluated using a transwell migration assay. Briefly, 5 × 10^4 ^cells were suspended in 100 μL of EBM-2 supplemented with 0.1% BSA and placed in the upper chamber of an 8.0-mm pore size transwell (Costar, Cambridge, MA). 600 μL of the final dilution was placed in the lower chamber. After incubation for 6 hours at 37°C in 5% CO_2_, the cells that had not migrated were removed from the upper surface of the filters using cotton swabs and those that migrated to the lower surface of the filters were fixed in methanol and stained with Giemsa's Stain Solution. Migration was determined by counting the cell number with a microscope. Five visual fields were randomly chosen for each assay. The average number of the migrating cells in these 5 fields was taken as the cell migration number of the group.

### Adhesion assay

EPCs were labeled with 4, 6-diamidino-2-phenylindole (Roche Applied Science, Indianapolis, IN). After detachment and centrifugation, EPCs were resuspended in adhesion buffer (0.5% bovine serum albumin in EBM-2) and identical numbers of cells were replated in fibronectin-coated 24-well culture plates, incubated for 60 min at 37°C, and then washed 3 times gently with adhesion buffer to remove the nonadherent cells. The adherent cells were counted by fluorescence microscopy at × 200 magnification. Five independent fields were assessed for each well, and the average number of adherent cells per × 200 field was determined.

To evaluate the ability of EPCs to adhere to confluent vascular endothelial cells, human vascular endothelial cells were cultured in 1640 medium containing 10% fetal bovine serum (Gibco) and served as a matrix for the adhesion assay. Briefly, a monolayer of vascular endothelial cells was prepared in 24-well culture plates 48 h before the assay. EPCs were labeled with 5, 6-carboxyfluorescein diacetate, succinimidyl ester (CFSE). Finally, EPCs were added to each well and incubated for 3 h at 37°C. Nonattached cells were gently removed with PBS and adherent cells were fixed with 4% paraformaldehyde and counted in five random fields under 200 × magnification. The results obtained revealed essentially unchanged results during a 4-week observational period with a variation coefficient less than 6%.

### Statistical analysis

Statistical analyses were performed with Statistical Package for Social Sciences 13.0 software program (SPSS, USA). The Mann-Whitney U test and Student's t-test were used to compare variables between the two groups. Multiple comparisons were analyzed by Anova followed by post-hoc analysis to adjust the significance level. Data are shown as means ± S.E. Statistical significance was considered as P < 0.05.

## Results

### Characterization of EPCs

**Figure 1:**
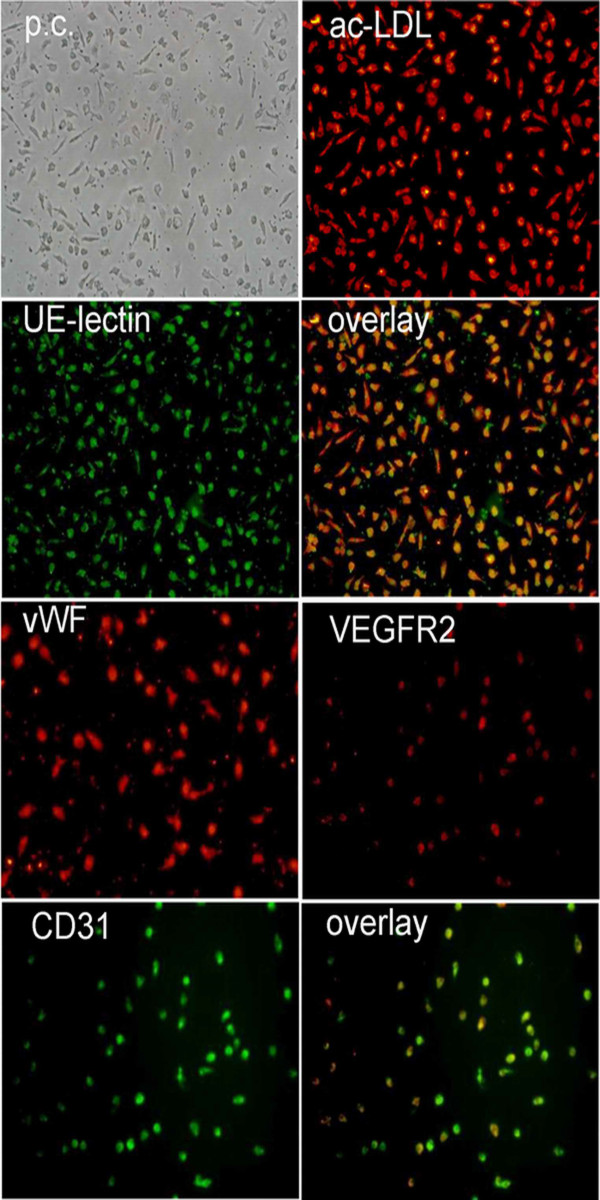
**Phenotypic characterization of EPCs from human peripheral blood**. After a week in culture, EPCs were stained with DIL-labeled ac-LDL, FITC-conjugated Ulex europaeus lectin and double stained with DIL-labeled ac-LDL and FITC-conjugated Ulex europaeus lectin (overlay). von Willebrand factor (vWF), VEGFR2, CD31 and double stained with VEGFR2 and CD31 (overlay) analysis were assessed by immunofluorescence (× 20).

After 7 days of culture, ex vivo expanded EPCs derived from peripheral blood of healthy human volunteers and ovarian cancer patients exhibited spindle-shaped morphology. EPCs were characterized as adherent and double positive for Dil-Ac-LDL uptake and lectin binding based on their appearance in a fluorescent microscope. A total of 93.8 ± 4.5% of adherent cells showed uptake of Dil-Ac-LDL and lectin binding after 7 days of culture in our study. The endothelial phenotype of these expanded EPCs was further characterized by the expression of endothelial markers such as von willebrand factor, CD31, and VEGFR2. Immunofluorescence showed that the cells were positive for vWF, CD31, and VEGFR2 (Figure. 1).

### Id1-RNAi-LV transfection inhibited the expression of Id1 mRNA and protein in EPCs

**Figure 2:**
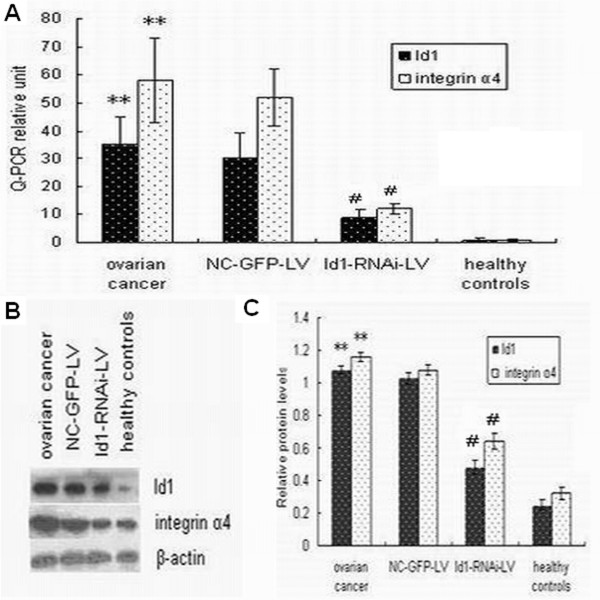
**Id1-RNAi-LV transfection silenced the mRNA and protein expression of Id1 and integrin α4 in EPCs**. (A) Id1 and integrin α4 mRNA expression by real-time RT-PCR. Data are expressed as means ± S.E. **p < 0.01 vs control, ^#^p < 0.05 vs ovarian cancer. (B) Typical Western blot images showing protein expression of Id1 and integrin α4 (β-actin is shown as a housekeeping control). (C) The graph showing the relative Id1 and integrin α4 protein levels normalized to β-actin. The results were expressed as the mean ± S.E.. **p < 0.01 vs. control, ^#^p < 0.05 vs. ovarian cancer.

We used real-time RT-PCR to examine mRNA expression of Id1 in EPCs of 25 patients with ovarian cancer, and Western blot analysis revealed a higher Id1 protein expression in human ovarian cancer EPCs than in 20 healthy controls. To confirm the role of Id1 in EPCs of patients with ovarian cancer we performed gene-silencing experiments. EPCs were transfected with lentiviral-Id1-siRNA vector. The negative control containing pGCSIL/U6 mock vector only was denoted NC-GFP-LV. After the Id1-RNAi-LV construct was transfected into EPCs of 6 patients with ovarian cancer, Id1 mRNA expression levels in transfected cells were compared to those in nontransfected and control-transfected (NC-GFP-LV) EPCs of 6 patients with ovarian cancer by quantitative RT-PCR. Cells with Id1-RNAi-LV transfection showed a 72% reduction in the level of Id1mRNA expression (Figure 2A). To further confirm the specificity of Id1-RNAi-LV-mediated Id1 silencing, Id1 protein expression was determined by Western blot. As shown in Figure 2B-C, Id1 protein expression in EPCs of 6 patients with ovarian cancer transfected with Id1-RNA-LV was significantly decreased compared to that in control EPCs. These results indicate that lentivirus-mediated RNAi was an effective way of modulating Id1 expression in cultured EPCs.

### Effects of Id1-RNAi-LV on EPCs proliferation, migration and adhesion

To examine EPCs functions in ovarian cancer we examined the proliferation, migration, and adhesion of EPCs. Proliferation, migration, and adhesion of EPCs are important for mobilization and recruitment. The EPCs of ovarian cancer patients showed increased migration and adhesion to fibronectin and endothelial cells. Statistical analyses revealed that ovarian cancer enhanced EPCs proliferation, migration, and adhesion.

**Figure 3:**
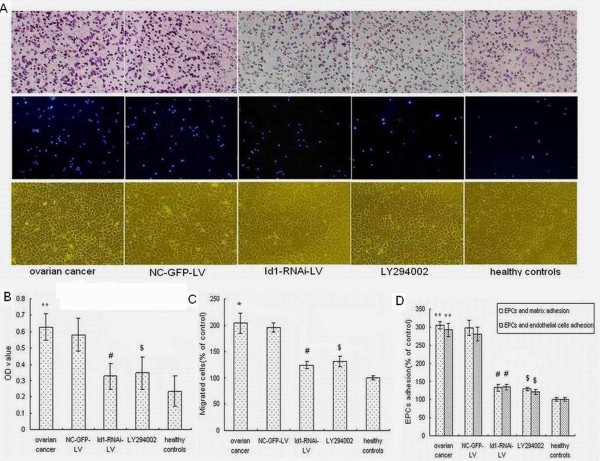
**Effects of Id1-RNAi-LV and LY294002 on EPCs proliferation, migration, and adhesion functions**. (A) Typical images of migration and adhesion as measured by transwell chamber and ECMatrix gel assay, respectively.  (B) Effects of Id1-RNAi-LV and LY294002 on EPCs proliferation. **p<0.01 vs. control, #,$p<0.05 vs. ovarian cancer.  (C-D) Accumulated data showing EPCs migration and adhesion functions. *p<0.05, **p<0.01 vs. control, ^#,$^p<0.05 vs. ovarian cancer.

After the Id1-RNAi-LV construct was transfected into EPCs, the cells were cultured for 7 days and we then performed EPCs proliferation, migration, and adhesion analysis. Id1-RNAi-LV markedly reduced EPCs functions. Cells transfected with Id1-RNAi-LV displayed less proliferation, migration, and adhesion abilities compared to non-transfected control cells, as shown in Figure 3A-D. Cells transfected with NC-GFP-LV, on the other hand, exhibited no change in cell proliferation, migration, and adhesion abilities, compared to non-transfected control EPCs. These data indicate that Id1 is crucial for the mobilization and recruitment of EPCs in ovarian cancer.

### Id1-RNAi-LV transfection inhibited the expression of integrin α4 mRNA and protein in EPCs

To explain the effect of Id1 on migration toward peripheral blood and recruitment to tumor tissues we explored the expression of integrin α4 on the EPCs surface of 25 patients with ovarian cancer. In keeping with the mRNA results, Western blots showed increased integrin α4 protein expression in EPCs.

After the Id1-RNAi-LV construct was transfected into EPCs of 6 patients with ovarian cancer, integrin α4 mRNA expression levels in transfected cells were compared to those in nontransfected and control-transfected (NC-GFP-LV) EPCs of 6 patients with ovarian cancer by quantitative RT-PCR. Cells with Id1-RNAi-LV transfection showed a 65% reduction in integrin α4 mRNA expression (Figure 2A). Integrin α4 protein expression was then determined by Western blot. As shown in Figure 2B, integrin α4 protein expression in EPCs transfected with Id1-RNA-LV was significantly decreased compared to that in control EPCs. These results indicate that the effect of Id1 on adhesion and angiogenesis is associated with activation of integrin α4.

### Effects of PI3K/Akt on Id1, integrin α4, and EPCs functions

**Figure 4:**
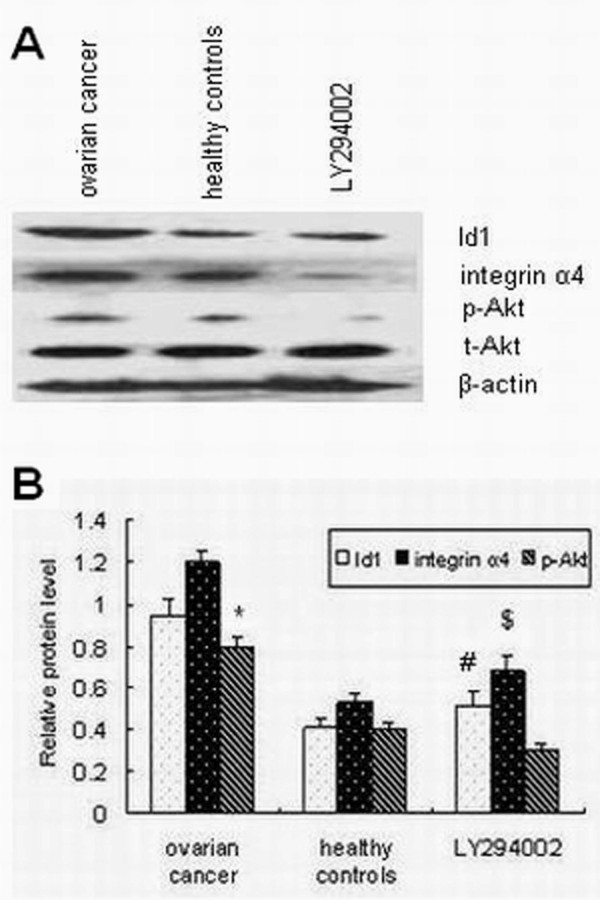
**Effects of PI3K/Akt on the protein expression of Id1, integrin α4, p-Akt in EPCs**. (A) Typical Western blot images showing protein expression of Id1, integrin α4, p-Akt and t-Akt (β-actin is shown as a housekeeping control). (B) The graph showing the relative Id1, integrin α4, and p-Akt protein levels normalized to actin. The results are expressed as the mean ± S.E. *p < 0.05 vs. control, ^#,$^p < 0.05 vs. ovarian cancer.

EPCs use a broad spectrum of mobilization and recruitment mechanisms to achieve with enhanced tumor metastasis (Shaked et al. 2006). To begin to determine which signaling transduction pathways might participate in Id1-mediated cell mobilization and recruitment in EPCs, we investigated PI3K/AKT pathway using pharmacological inhibitors. Elevated AKT-Ser473 phosphorylation was observed in EPCs, and it was completely abolished by LY294002 (1 μmol/L). As Figure 4A-B shows, Id1 and integrin α4 expression were also strongly decreased by LY294002. Figure 3A-D show that EPCs of patients with ovarian cancer had increased levels of cell proliferation, migration and adhesion, which were strongly decreased by incubation with LY294002. These results indicate that Id1-induced EPCs mobilization and recruitment is mediated by the PI3K/AKT pathway. Thus Id1 induced migration and adhesion of EPCs is mediated by an increase in integrin α4 expression and is regulated by the PI3K/Akt pathway.

## Discussion

Ovarian cancer is one of the most aggressive gynecological malignancies and its high mortality is most often a direct result of delays in diagnosis. Only 25% of ovarian cancers are diagnosed while the malignancy is still confined to the ovary, and the cure rate in these patients can reach 90%. The remaining 75% of ovarian tumors have spread beyond the ovary by the time of diagnosis and the cure rate for these patients is less than 20% [11]. With the advent of molecular-targeted therapies, treatment for ovarian cancer is now moving beyond conventional chemotherapy. Inhibition of the specific cytokines essential for tumor vascularization is such a therapy [12]. Thus anti-angiogenesis therapy has become a new strategy for ovarian cancer treatment [13].

Evidence continues to accumulate confirming the importance of EPCs in the neovascularization of tumor tissues. Many researchers have focused on EPCs biology and potential clinical applications [14-16]. Previous studies have suggested that bone marrow-derived EPCs can migrate to tumor foci and promote tumor growth and metastasis. However, the signals that mediate mobilization and recruitment of these cells to tumors are not well understood. We showed previously that EPCs numbers are increased in ovarian cancer patients and EPCs numbers were related to tumor progression and angiogenesis. Here, using blood samples from 25 ovarian cancer patients, we demonstrated that Id1 is involved in enhancing EPCs migration and adhesion. Id1 may mediate EPCs mobilization and recruitment to ovarian cancer tissues. We further explored the effect of Id1 and related signaling pathways on EPCs of patients with ovarian cancer.

Id1 has been implicated in a variety of cellular processes including cell growth, differentiation, angiogenesis, and neoplastic transformation. Id1 is expressed in various tumor tissues and cells [17]. Id1 knockout mice were critical in demonstrating that BM-derived progenitors are the source of tumor endothelium in some tumor types and grades, as Id1 knockout mice failed to mobilize these progenitors and transplantation of Id1 knockout mice with wild type BM was shown to rescue the observed vascular defects [8,18]. The importance of Id1+ progenitor cells in vascular rebound was confirmed recently by results from vascular disrupting therapies [19]. The mechanism by which Id1 controls the generation of EPCs is also beginning to be explored. In recent studies, Id1 was shown to be expressed in long term repopulating hematopoietic stem cells (lin- Sca+ kit+ CD34-) in the BM and Id1 loss was shown to lead to an upregulation of the expression of the cyclin-dependent kinase inhibitor p21. The expression of p21 in turn drives the stem cells towards a more committed myeloid state, as assessed by gene expression profiling; this myeloid differentiation is associated with the depletion of cells capable of endothelial cell fate commitment [20,21]. These results suggest that Id1 is required in early hematopoietic stem cells to restrain the commitment to the myeloid lineage and preserve a pool of cells that give rise to endothelial progenitors in response to vasculogenic growth signals. Although Id1 and Id3 are thought to be functionally redundant in many cell types [1,22]. It is not yet known if this is the case in EPCs.

In this study we show an essential role for Id1 in EPCs mobilization and recruitment to the tumor. Our real-time RT-PCR analysis indicates that Id1 expression in ovarian cancer is significantly increased compared to that in healthy subjects. Importantly, EPCs isolated from ovarian cancer patients display increased integrin α4 expression and baseline migration and adhesion. This is interesting in light of a recent report showing that integrin α4 promotes the homing of circulating progenitor cells and other bone marrow-derived mononuclear circulating cells not only to tumor tissues but also to inflamed and ischemic tissues [23]. To determine the role of Id1 in EPCs, we repressed its expression. As expected, transfection of EPCs with Id1-special siRNA silenced Id1 expression and abolished upregulation of integrin α4. Furthermore, migration and adhesion were both inhibited in EPCs when they were transduced with Id1-RNAi-LV. This indicates that Id1 is important for EPCs migration and adhesion. Our data demonstrate that down-regulation of Id1 may reduce the expression of integrin α4, thereby contributing to the functional inhibition we observed in our study.

Little is known about the molecular signaling pathways underlying EPCs mobilization and recruitment, particularly in ovarian cancer patients. It has been shown that Id1 is an important regulator for the fate of hematopoietic stem cells. In this study, we showed that high Id1 in EPCs is mediated by the PI3K/Akt pathway, a key pathway in mediating vasculogenesis. We also demonstrated that ovarian cancer activated the PI3K/Akt signaling pathway in EPCs. This is a novel finding. Therefore, we concluded that signaling through the P13K/Akt signaling pathway upregulates Id1, which enhances mobilization and recruitment via integrin α4. We showed that Akt is phosphorylated in EPCs of ovarian cancer patients, and inhibition of PI3K/Akt downregulated the expression level of Id1 and integrin α4 and reduced EPCs functions. Taken together, our data support the notion that ovarian cancer EPCs migration and recruitment via the PI3K/Akt-Id1- integrin α4 signaling pathway may be responsible for increased EPCs levels in ovarian cancer.

## Conclusions

We showed that Id1 induced proliferation, migration and adhesion of human EPCs via the PI3K and Akt signaling pathway. Our data also provide further insights into the understanding of neovascularization. These findings raise the possibility that therapeutic strategies aiming to reduce EPCs angiogenesis have a two pronged effect: they might enhance the efficacy of certain cytotoxic anti-angiogenesis ovarian cancer therapies while at the same time reducing the risk of ovarian cancer metastases.

## Competing interests

The authors declare that they have no competing interests.

## Authors' contributions

YS participated in study design, carried out most of the experiments, and drafted the manuscript. LZ participated in collecting samples and manuscript preparation. QW conceived of the study, and participated in its design and coordination. JB assisted with cell culture. ZC participated in study design and statistical analysis. AL assisted with the critical revision of the manuscript. All authors read and approved the final manuscript.

## Pre-publication history

The pre-publication history for this paper can be accessed here:

http://www.biomedcentral.com/1471-2407/10/459/prepub

## References

[B1] Lyden D Young AZ Zagzag D Yan W Gerald W O'Reilly R Bader BL Hynes RO Zhuang Y Manova K Benezra R Id1 and Id3 are required for neurogenesis, angiogenesis and vascularization of tumour xenografts Nature 1999 401 6754 670 677 10.1038/44334 10537105

[B2] Bertolini F Shaked Y Mancuso P Kerbel RS The multifaceted circulating endothelial cell in cancer: towards marker and target identification Nature Reviews Cancer 2006 6 11 835 845 10.1038/nrc1971 17036040

[B3] Young PP Vaughan DE Hatzopoulos AK Biologic properties of endothelial progenitor cells and their potential for cell therapy Prog Cardiovasc Dis 2007 49 6 421 429 10.1016/j.pcad.2007.02.004 17498522 PMC1978244

[B4] Li B Sharpe EE Maupin AB Teleron AA Pyle AL Carmeliet P Young PP VEGF and PlGF promote adult vasculogenesis by enhancing EPC recruitment and vessel formation at the site of tumor neovascularization FASEB J 2006 20 9 1495 1497 10.1096/fj.05-5137fje 16754748

[B5] Stoelting S Trefzer T Kisro J Steinke A Wagner T Peters SO Low-dose oral metronomic chemotherapy prevents mobilization of endothelial progenitor cells into the blood of cancer patients In Vivo 2000 822 6 831 836 19181016

[B6] Perk Jonathan Gil-Bazo Ignacio Chin Yvette de Candia Paola Chen John JS Zhao Yuntao Chao Shirley Cheong Wai Ke Yaohuang Al-Ahmadie Hikmat Gerald William L Brogi Edi Benezra Robert Reassessment of Id1 protein expression in human mammary, prostate, and bladder cancers using a monospecific rabbit monoclonal anti-Id1 antibody Cancer Res 2006 66 22 10870 10877 10.1158/0008-5472.CAN-06-2643 17108123

[B7] Shaked Y Ciarrocchi A Franco M Lee CR Man S Cheung AM Hicklin DJ Chaplin D Foster FS Benezra R Kerbel RS Therapy-induced acute recruitment of circulating endothelial progenitor cells to tumors Science 2006 313 5794 1785 1787 10.1126/science.1127592 16990548

[B8] Gao D Nolan DJ Mellick AS Bambino K McDonnell K Mittal V Endothelial progenitor cells control the angiogenic switch in mouse lung metastasis Science 2008 319 5860 195 198 10.1126/science.1150224 18187653

[B9] Maw MK Fujimoto J Tamaya T Overexpression of inhibitor of DNA-binding (ID)-1 protein related to angiogenesis in tumor advancement of ovarian cancers BMC Cancer 2009 10 9 430 10.1186/1471-2407-9-430 PMC2796680 20003244

[B10] Zhang X Ling MT Feng H Wong YC Tsao SW Wang X Id-I stimulates cell proliferation through activation of EGFR in ovarian cancer cells Br J Cancer Dec 2004 91 12 2042 2047 10.1038/sj.bjc.6602254 PMC2409798 15599381

[B11] Lorusso D Ferrandina G Graggi S Gadduci A Pignata S Tateo S Bia-monte R Manzione L Di Vagno G Ferrau F Scambia G Phase III multicenter randomized trial of amifostine as cytoprotect-ant in first-line chemotherapy in ovarian cancer patients Ann Oncol 2003 14 7 1086 1093 10.1093/annonc/mdg301 12853351

[B12] Melichar B Urbanek L Krcmova L Kalabova H Svobodova I Drag-ounova E Vesely P Hyspler R Solichova D Urinary neopterin in patients with ovarian cancer Pteridines 2006 17 4 145 153

[B13] Spannuth WA Sood AK Coleman RL Angiogenesis as a strategic target for ovarian cancer therapy Nat Clin Pract Oncol 2008 5 4 194 204 10.1038/ncponc1051 18268546

[B14] Khan ZA Melero-Martin JM Wu X Paruchuri S Boscolo E Mulliken JB Bischoff J Endothelial progenitor cells from infantile hemangioma and umbilical cord blood display unique cellular responses to endostatin Blood 2006 108 3 915 921 10.1182/blood-2006-03-006478 16861344 PMC1895853

[B15] Dome B Timar J Dobos J Meszaros L Raso E Paku S Kenessey I Ostoros G Magyar M Ladanyi A Bogos K Tovari J Identification and clinical significance of circulating endothelial progenitor cells in human non-small cell lung cancer Cancer Res 2006 66 14 7341 7347 10.1158/0008-5472.CAN-05-4654 16849585

[B16] Khoo CP Pozzilli P Alison MR Endothelial progenitor cells and their potential therapeutic applications Regen Med 2008 3 6 863 876 10.2217/17460751.3.6.863 18947309

[B17] de Candia P Benera R Solit DB A role for Id proteins in mammary gland physiology and tumorigenesis Adv Cancer Res 2004 92 81 94 full_text 15530557 10.1016/S0065-230X(04)92004-0

[B18] Shaked Yuval Ciarrocchi Alessia Franco Marcela Lee Christina R Man Shan Cheung Alison M Hicklin Daniel J Chaplin David Stuart Foster F Benezra Robert Kerbel Robert S Therapy-induced acute recruitment of circulating endothelial progenitor cells to tumors Science 2006 313 5794 1785 1787 10.1126/science.1127592 16990548

[B19] Lyden D Hattori K Dias S Costa C Blaikie P Butros L Chadburn A Heissig B Marks W Witte L Wu Y Hicklin D Zhu Z Hackett NR Crystal RG Moore MA Hajjar KA Manova K Benezra R Rafii S Impaired recruitment of bone-marrow-derived endothelial and hematopoietic precursor cells blocks tumor angiogenesis and growth Nat Med 2001 7 11 1194 1201 10.1038/nm1101-1194 11689883

[B20] Ciarrocchi A Jankovic V Shaked Y Nolan DJ Mittal V Kerbel RS Nimer SD Benezra R Id1 restrains p21 expression to control endothelial progenitor cell formation PLoS One 2007 2 12 e1338 10.1371/journal.pone.0001338 18092003 PMC2129121

[B21] Qian Y Chen X ID1, inhibitor of differentiation/DNA binding, is an effector of the p53-dependent DNA damage response pathway J Biol Chem 2008 283 33 22410 22416 10.1074/jbc.M800643200 18556654 PMC2504896

[B22] Benezra R Rafii S Lyden D The Id proteins and angiogenesis Oncogene 2001 20 58 8334 8341 10.1038/sj.onc.1205160 11840326

[B23] Jin Hui Aparna Aiyer Su Jingmei Per Borgstrom Dwayne Stupack Martin Friedlander Varner Judy A homing mechanism for bone marrow-derived progenitor cell recruitment to the neovasculature The Journal of Clinical Investigation 2006 116 3 652 662 10.1172/JCI24751 16498499 PMC1378185

